# Professor Halford

**Published:** 1910-08-20

**Authors:** 


The death of Professor Halford, first principal of the Medical School at the University of Melbourne, occurred on May 27. Although he retired 14 years ago, he was not forgotten as one of the few remaining pioneers of the university, which he joined in 1862. He left a large number of distinguished pupils, amongst them the present Dean of the Faculty of Medicine (Professor Allen), Dr. B. Poulton (lecturer on surgery at Adelaide), and Dr. F. C. Madden (professor of surgery at Cairo). Professor Halford was the author of a system of snake-bite cure discovered from his close study of the symptoms and blood changes that occur from poisoning by snake-bite. He was born in 1824. Of his six sons, two have followed their father's profession—Dr. George Billing Halford, of Malvern, near Melbourne, and Dr. Arthur Halford, of Brisbane, Queensland.

